# Investigating Why Comparing Intravenous and Perineural Dexamethasone Is Scientifically Unfounded

**DOI:** 10.7759/cureus.98950

**Published:** 2025-12-11

**Authors:** Kartik Sonawane

**Affiliations:** 1 Anesthesiology, Ganga Medical Centre and Hospitals Pvt. Ltd., Coimbatore, IND

**Keywords:** block prolongation, dexamethasone, eras, intravenous, neuroprotection, perineural, perineural adjuvants, pharmacokinetics, regional anesthesia

## Abstract

Dexamethasone is a widely used adjuvant in regional anesthesia, yet debate persists over whether intravenous (IV) and perineural routes are interchangeable. This editorial argues that head-to-head comparisons are mechanistically unsound and clinically misleading. IV dexamethasone distributes systemically after cardiopulmonary transit, yielding low, variable target-site bioavailability at the nerve; systemic anti-inflammatory and antiemetic effects within enhanced recovery pathways dominate its benefits. Perineural dexamethasone creates a high-concentration depot at the neural interface, prolonging block reliability through reduced vascular uptake and a locally anti-inflammatory microenvironment, while conferring neuroprotection via antiedema, antifibrotic, microcirculatory, and antineurotoxic actions. Published studies reporting either superiority of perineural dosing or equivalence between routes likely reflect heterogeneity in populations, procedures, timing, anesthetic strategies, and adjuvant regimens rather than true pharmacologic parity. Methodological parity of milligram dosing across routes is a key flaw; concentration- or exposure-matched designs are needed. Clinically, IV dexamethasone should be used for systemic modulation (including postoperative nausea and vomiting reduction), whereas perineural dexamethasone should be selected to extend block duration and protect the nerve; combined use can be synergistic when both aims matter. Reframing the debate from competition to context-specific integration aligns practice with pharmacology and may improve patient-centered outcomes.

## Editorial

Introduction

Dexamethasone is widely regarded as a reliable adjuvant in regional anesthesia (RA) owing to its anti-inflammatory, antiemetic, and block-prolonging properties. However, an ongoing debate centers on whether intravenous (IV) or perineural administration is superior for improving the quality and duration of peripheral nerve blocks. Though both utilize the same pharmacological compound, equating the two routes as functionally interchangeable is an oversimplification. In truth, these two approaches are distinct in their pharmacokinetics, pharmacodynamics, and clinical objectives. This editorial explains why IV-perineural comparisons are mechanistically unsound and clinically misleading, and it advocates route-specific, context-driven use.

A tale of two lights: conceptual distinction

**Figure 1:**
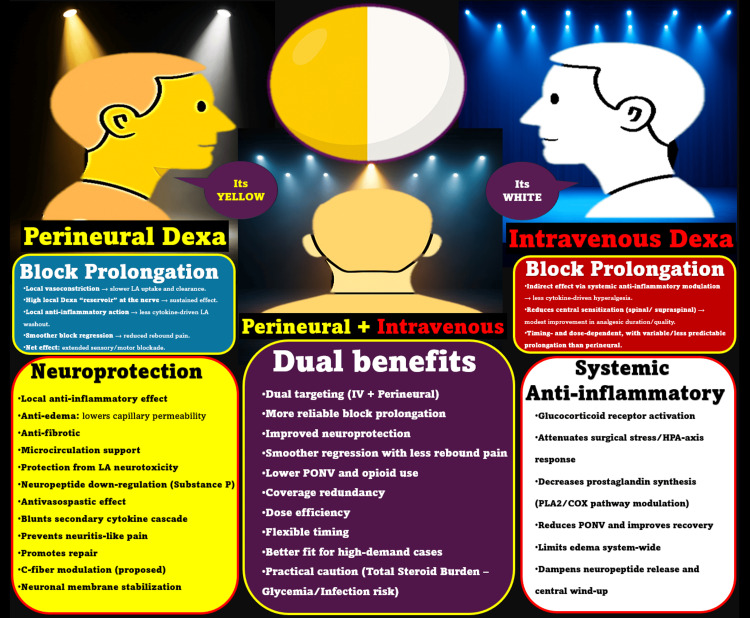
Clinician’s perspective on route-dependent effects of dexamethasone. The two-toned sphere symbolizes distinct, non-interchangeable routes that reveal different halves of the truth depending on the viewer’s position. The spotlights extend the metaphor: the focused yellow beam represents perineural, nerve-targeted action, while the ambient white/blue wash represents IV, systemic modulation. The center view integrates both, illustrating how seeing both halves under both lights conveys the complete, complementary picture of dexamethasone’s route-dependent effects. Sphere color coding: yellow, perineural/local; white, IV/systemic. Dexa: dexamethasone; IV: intravenous; LA: local anesthetic; PONV: postoperative nausea and vomiting; HPA: hypothalamic-pituitary-adrenal. Source: this figure was created by the author, KS.

To conceptualize the difference, imagine dexamethasone as light (Figure 1). When administered intravenously, it produces an effect akin to a soft, ambient glow, illuminating the entire systemic landscape with broad anti-inflammatory benefits. It calms the hypothalamic-pituitary-adrenal axis, reduces postoperative nausea, and supports stress modulation, thereby benefiting overall perioperative recovery as part of enhanced recovery after surgery protocols [1-3].

In contrast, perineural dexamethasone functions as a focused spotlight, sharpened and site-specific. It targets the site of nerve injury or blockade with precision, saturating the perineural environment with potent anti-inflammatory and neuroprotective effects [4]. These two lighting modes, ambient and focused, do not compete but serve different roles. Their simultaneous use represents a synergistic model of care, illuminating both the body and the periphery with targeted intent.

Pharmacokinetics: one drug, two journeys

The two administration routes follow entirely different pharmacokinetic pathways. IV dexamethasone undergoes systemic dilution after traversing the right heart, lungs, left heart, and high-perfusion organs before finally reaching peripheral nerve tissues, which are low-perfusion zones. This pattern reflects perfusion-limited distribution: after an IV bolus, the drug partitions first into highly perfused compartments with slower equilibration into low-perfusion tissues such as perineural connective planes. By the time it reaches the nerve, its bioavailability is minimal. Its impact is therefore indirect and lacks consistency in enhancing nerve block duration.

Perineural dexamethasone, however, is delivered directly to the site of action. It forms a high-concentration depot around the nerve, evading first-pass metabolism and systemic dilution. It reduces vascular absorption through localized vasoconstriction, prolongs the dwell time of local anesthetics (LAs), and creates a favorable neurochemical microenvironment that supports longer block duration and improved analgesia. Taken together, the depot plus diffusion/uptake-limited egress support higher and more sustained perineural exposure than after IV dosing [4]. Direct head-to-head quantification at the neural interface is limited and beyond the scope of this editorial.

Mechanism of action: block prolongation and neuroprotection

Regardless of the route, dexamethasone acts as a glucocorticoid receptor agonist, modulating the transcription of proinflammatory cytokines such as interleukin-1, interleukin-6, and tumor necrosis factor-α [5]. However, only perineural dexamethasone leverages localized mechanisms to a therapeutic advantage. Perineural dexamethasone acts directly on the unmyelinated C-fibers, inhibits potassium channels involved in nociceptive signaling, stabilizes nerve membranes, and suppresses local neurogenic inflammation [6-9]. Local vasoconstriction slows systemic uptake, sustaining perineural levels; concomitant suppression of substance P dampens nociceptive input [10]. Together, these effects prolong sensory and motor blockade, reduce rebound pain, smooth regression, and improve overall block quality [11].

Conversely, IV dexamethasone contributes to global anti-inflammatory signaling and suppresses central sensitization, but does not exert focused perineural effects. Its systemic spread and protein binding reduce the availability of neural tissue, making it less reliable for prolonging the block.

Different clinical objectives

The intent behind each route underscores its non-interchangeability. IV dexamethasone is primarily used to reduce postoperative nausea, systemic inflammation, and stress, aims that are holistic but generalized. In contrast, perineural dexamethasone is tailored for one precise goal: to improve the duration and quality of regional nerve blocks. This objective is narrow, measurable, and directly aligned with the goals of RA. Thus, any attempt to compare these two modalities as equivalents neglects the distinct purposes they serve. It creates a false dichotomy and risks misleading practitioners about the optimal use of dexamethasone in different clinical contexts. Conflating these distinct objectives creates a false equivalence and risks misapplication in practice. Key distinctions between routes and their combined use are summarized in Table 1.

**Table 1:** Comparative overview of perineural versus intravenous dexamethasone and their synergistic application. HPA: hypothalamic-pituitary-adrenal; IL-1/IL-6: interleukin-1/interleukin-6; TNF-α: tumor necrosis factor-alpha; PONV: postoperative nausea and vomiting; ERAS: enhanced recovery after surgery. Source: this table was created by the author, KS, synthesizing concepts from references [1-15]; supporting details are explained in the main text.

Aspect	Perineural dexamethasone	Intravenous dexamethasone	Combined use (synergistic)
Primary objective	Local block prolongation and neuroprotection	Systemic anti-inflammatory and antiemetic effect	Optimize both regional block efficacy and systemic recovery
Site of action	Directly near the peripheral nerves	Central nervous system and systemic circulation	Concurrent peripheral and central modulation
Mechanism of action	Inhibits nociceptive C-fiber transmission, stabilizes axonal membranes, and suppresses perineural inflammation	Blunts HPA axis, reduces systemic cytokines (IL-1, IL-6, TNF-α), antiemetic action	Dual pathway activation: targeted neural and systemic anti-inflammatory synergy
Block duration prolongation	Pronounced and dose-dependent	Mild and variable	Additive or prolonged effect with dual administration
Neuroprotection	Anti-inflammatory, antifibrotic, antioxidative, and microcirculatory enhancement	No direct neuroprotection at the nerve level	Preserves block quality while ensuring systemic stress reduction
Anti-inflammatory action	Local suppression of inflammatory mediators	Broad cytokine modulation and immune suppression	Comprehensive control of both local and systemic inflammation
Impact on PONV	No intrinsic antiemetic benefit	Strong antiemetic effect; standard for PONV prevention	Retains antiemetic protection with extended analgesia
Rebound pain prevention	Smoother regression, avoids local inflammatory flares	Higher risk due to lack of local modulation	Reduced rebound risk when perineural component included
Pharmacokinetics	High local concentration, minimal systemic dilution	Extensive systemic distribution, high protein binding	Combines site-specific depot with systemic bioavailability
Dose consideration	Lower dose effective at the site of action	Requires higher systemic dose	Requires individualized titration for optimal balance
Limitations	Off-label use in some regions; institutional policies may restrict	May inadequately prolong block duration if used alone	Demands tailored integration and clinical judgment
Ideal use case	High-risk nerve-dense blocks (e.g., brachial plexus, sciatic), concern for neural injury	High risk of PONV, systemic inflammation, or part of ERAS pathway	Surgeries requiring prolonged analgesia + systemic modulation (e.g., major joint replacements, thoracic surgeries)

Literature conflicts: perspective shapes interpretation

Multiple published studies have weighed in on this debate, often with contrasting conclusions. Some studies report longer block duration with perineural versus IV dexamethasone [12,13], whereas others find equivalent analgesic efficacy and safety between routes [14]. Such discrepancies are not merely academic; they stem from heterogeneity in study design, patient populations, types of surgery, timing of block placement (pre- vs. postoperative), anesthesia modalities (general, spinal, or sedation-based), and varying LA combinations and doses. In this fragmented landscape, it is easy for a clinician to find literature that supports either stance, and many adopt a perspective aligned with their routine practice or local institutional norms.

The two-toned sphere (Figure 1) aptly symbolizes this divergence in interpretation. One clinician, standing on the left, sees only the yellow (perineural) side and argues for its superiority. Another, on the right, views only the white (IV) hemisphere and supports systemic use. Both are correct in their own context, but only the third observer, standing at the front, sees the whole truth: each route has distinct utilities, and their combination offers additive value. Therefore, reducing this conversation to a binary “IV vs. perineural” debate misses the larger pharmacological and clinical nuance. We must shift our lens from “either-or” to “when and how,” and approach dexamethasone usage not as a competition between routes, but as an opportunity for rational integration based on context, patient goals, and block strategy.

Neuroprotective edge of perineural dexamethasone

First, it exerts a potent anti-inflammatory effect by suppressing the release of inflammatory mediators, thereby limiting perineural edema and tissue injury [4]. Second, it reduces capillary leakage, thereby diminishing the risk of nerve compression from localized swelling. Third, its antifibrotic properties inhibit fibroblast proliferation and collagen deposition, reducing the risk of postoperative adhesions and nerve tethering [15,16]. Additionally, dexamethasone stabilizes neuronal membranes, reducing ectopic firing and neuropathic pain, and enhances perineural microcirculation by limiting inflammation and edema, thereby improving oxygenation, preserving axonal transport, and supporting recovery in high-risk regions such as the supraclavicular fossa [17].

Crucially, it mitigates LA-induced neurotoxicity by attenuating oxidative stress, mitochondrial injury, and apoptosis [4]. Furthermore, perineural dexamethasone helps prevent neuritis-like pain syndromes by dampening neuropeptide release, such as substance P, and breaking the cycle of peripheral and central sensitization [10]. It offers smoother block regression without rebound pain or inflammatory flares, an important feature in patient comfort and satisfaction. In minor subclinical nerve injuries, it supports endoneurial flow, axonal transport, and remyelination, potentially accelerating recovery [4,17]. These advantages cannot be matched by IV dexamethasone, which exerts no direct influence on perineural inflammation or perfusion. Given this spectrum of actions, it is evident that perineural dexamethasone does more than simply prolong blocks; it actively safeguards the neural interface. Therefore, substituting IV dexamethasone in its place not only compromises efficacy but also forfeits its protective benefits.

Dose mismatch: a methodological oversight

Another critical issue is the false equivalence of dosing. The literature continues to present direct comparisons between these two routes, often using equal milligram doses without accounting for bioavailability or site-specific pharmacology. This approach introduces serious methodological flaws. A 4 mg dose of IV dexamethasone does not equate to 4 mg delivered perineurally in terms of nerve-site exposure, duration of effect, or physiological impact. Model-based work has estimated that ~8 mg IV may approximate the block-prolonging effect of ~4 mg perineural for selected outcomes, but such equipotency estimates reflect duration modeling, not pharmacologic interchangeability [18]. Such study designs risk distorting clinical guidelines and creating confusion in anesthetic practice, particularly in institutions that discourage off-label perineural use without appreciating the differential outcomes. Where perineural dexamethasone is considered off-label, clinicians should weigh regulatory context against mechanistic plausibility and clinical benefit. Future work should use exposure-matched or concentration-informed designs rather than milligram-to-milligram parity.

Ethical and clinical imperatives

Although the reported incidence of nerve injury is low (~0.4%), its consequences are devastating [19]. A single nerve injury can result in lifelong disability and pain. A single misdirected injection or missed opportunity for neuroprotection can result in permanent loss of function, chronic pain, and devastating consequences for the patient and their family. Preventive strategies that reduce nerve inflammation, edema, and fibrosis, such as perineural dexamethasone, carry moral weight. Ignoring its neuroprotective role because of flawed comparisons is not just a scientific oversight; it is an ethical one. Every block represents a sacred trust between patient and clinician. Optimizing safety and efficacy with evidence-based strategies, including perineural adjuvants, honors that trust.

Conclusion: shift from competition to complementarity

In conclusion, intravenous and perineural dexamethasone are not interchangeable modalities but rather complementary tools with distinct roles in perioperative management. Their pharmacokinetic profiles, mechanisms of action, and clinical purposes diverge so substantially that direct comparisons are not only unscientific but potentially harmful to evidence-based practice. Rather than frame them in opposition, we should adopt a “contextual synergy” model: IV dexamethasone optimizing systemic recovery and antiemetic effect, and perineural dexamethasone enhancing nerve block efficacy and safety. As RA continues to evolve, clarity about the distinct functions of each route will allow clinicians to tailor strategies that balance scientific evidence and patient well-being.
